# Curricular Analysis of Digital Health and Health Informatics in Medical Colleges Across Saudi Arabia

**DOI:** 10.7759/cureus.66892

**Published:** 2024-08-14

**Authors:** Anas Alhur

**Affiliations:** 1 Health Informatics, University of Hail College of Public Health and Health Informatics, Hail, SAU

**Keywords:** vision 2030, saudi arabia, curriculum analysis, medical education, health informatics, digital health

## Abstract

Introduction

The integration of digital health and health informatics into medical education is essential for preparing future healthcare professionals in the rapidly evolving healthcare landscape. Digital health encompasses various technologies, including electronic health records, telemedicine, mobile health applications, and data analytics, all transforming healthcare delivery and management. Health informatics focuses on the efficient use of information technology to support and improve healthcare services. This study aims to analyze the extent to which digital health and health informatics are integrated into the study plans of medical colleges across Saudi Arabia, in alignment with the national goals outlined in Vision 2030.

Methods

A document analysis methodology was employed to review study plans and curricula from a purposive sample of medical colleges in Saudi Arabia. The sample included both public and private institutions such as King Abdul Aziz University, University of Bisha, Dar Al Uloom University, and Princess Nourah bint Abdulrahman University. Data were collected from official university websites, academic catalogs, and through direct communication with university departments. Key information regarding course offerings, credit hours, course content, and objectives related to digital health and health informatics was extracted and recorded in a structured format.

Results

The analysis revealed that only a few medical colleges in Saudi Arabia have integrated dedicated courses on digital health and health informatics into their curricula. For instance, Dar Al Uloom University, Princess Nourah bint Abdulrahman University, and University of Hail offer specific courses on medical informatics. However, the majority of the institutions either lack such courses or offer them in a limited capacity. The extent and depth of coverage of these courses vary widely among the institutions that offer them.

Conclusion

The study highlights the need for a more standardized and comprehensive approach to integrating digital health and health informatics into the medical curricula of Saudi Arabian universities. Recommendations include expanding course offerings, developing standardized curricula, investing in faculty development, and utilizing advanced technologies. Addressing these gaps will better prepare medical graduates for the modern, technology-driven healthcare environment and align with the national objectives of Vision 2030.

## Introduction

The integration of digital health and health informatics into medical education is increasingly recognized as essential for preparing future healthcare professionals. Digital health encompasses a wide range of technologies, including electronic health records (EHRs), telemedicine, mobile health applications, and data analytics, which are transforming healthcare delivery and management. Health informatics focuses on the efficient use of information technology to support and improve healthcare services. As these fields evolve, medical curricula must adapt to ensure that graduates are equipped with the necessary knowledge and skills.

Globally, the inclusion of digital health and health informatics in medical education has gained significant attention. Studies indicate that exposure to these fields during medical training enhances students' competencies in utilizing health information systems, improves clinical decision-making, and fosters innovation in healthcare delivery [1-2]. The World Health Organization has advocated for the integration of digital health education, highlighting its potential to bridge knowledge gaps and improve healthcare outcomes [3].

In the Middle East, there has been a concerted effort to modernize medical education by incorporating digital health competencies. A study demonstrated that medical students who received training in health informatics showed improved proficiency in managing patient data and using telemedicine platforms [4]. Similar initiatives have been reported in the United Arab Emirates and Qatar, where medical schools have revamped their curricula to include courses on health informatics and digital health technologies [5].

Saudi Arabia's Vision 2030 outlines a strategic framework to transform the nation's economy, with a significant focus on healthcare reform. The Ministry of Health has launched various initiatives to digitize healthcare services, including the National eHealth Strategy, which aims to establish a comprehensive electronic health ecosystem [6]. In line with these national objectives, Saudi medical colleges are increasingly recognizing the importance of integrating digital health and health informatics into their curricula.

Despite the national emphasis on digital health, there is limited research on the actual integration of these topics in Saudi medical education. A survey of medical schools in Saudi Arabia found that while some institutions have introduced courses on health informatics, the extent and depth of coverage vary widely. This variability suggests an inconsistency in how these crucial subjects are being taught, potentially affecting the quality of education that students receive. The study highlighted a need for standardized curricula and more comprehensive training programs to ensure all students gain the necessary competencies [7]. Another study revealed that many students experienced psychological pressure and a lack of understanding of the field, further indicating a need for better curriculum alignment and support structures to help students cope with the demands of this emerging discipline [8].

Implementing digital health and health informatics education in Saudi medical colleges faces several challenges, including a lack of qualified faculty, limited resources, and resistance to curriculum changes [9-11]. However, there are also significant opportunities. Collaboration with international institutions, investment in faculty development, and leveraging technology can facilitate the integration of these critical areas into medical education.

The primary aim of this study is to analyze the extent and manner in which digital health and health informatics are integrated into the study plans of medical colleges across Saudi Arabia. This analysis seeks to evaluate the current state of these curricula, identify gaps and challenges, and provide recommendations for enhancing the educational frameworks to better prepare future healthcare professionals.

## Materials and methods

Data collection

Document Analysis

This section details the methodology used for collecting and analyzing study plans and curricula from a purposive sample of medical colleges in Saudi Arabia to evaluate the integration of digital health and health informatics.

Sample Selection

A purposive sampling technique was employed to select medical colleges known for their diverse and comprehensive medical education programs. The sample included both public and private institutions to provide a well-rounded analysis. The institutions selected for this study included King Abdul Aziz University, University of Bisha, Dar Al Uloom University, Princess Nourah bint Abdulrahman University, and other notable medical colleges across Saudi Arabia.

Data sources

The study plans and curricula were primarily sourced from the (1) official websites of the selected universities, where available. In addition, detailed (2) academic catalogs were reviewed to extract information on course offerings related to digital health and health informatics. When online resources were insufficient, (3) direct communication with university departments was undertaken to request the most recent and comprehensive curricula and study plans.

Data extraction process

The data extraction process involved several key steps. First, study plans and curricula were collected from the selected institutions using official university websites, academic catalogs, and direct communication with university departments. Second, an initial review of the gathered documents was conducted to identify sections relevant to digital health and health informatics. Third, these relevant sections were then examined in detail, focusing on extracting course titles, codes, credit hours, and detailed course descriptions. Fourth, the extracted information was systematically recorded in a structured format to enable comparison across different institutions. Finally, to ensure accuracy and completeness, the recorded information was cross-checked against the original documents, with clarifications sought from university departments when necessary.

Detailed methodology steps

First, medical colleges were selected based on their reputation, availability of comprehensive medical programs, and diversity in public and private status. Second, official university websites were accessed, and available academic catalogs and study plans were downloaded. Where online information was insufficient, relevant university departments were contacted via email or phone to request the most recent and comprehensive curriculum documents. Third, the gathered documents were skimmed to locate relevant sections on digital health and health informatics courses. The structure of each document was noted to facilitate the systematic extraction of relevant data. Fourth, for each identified course, the course title, code, credit hours, and a detailed description of the course content and objectives were recorded. The focus was on extracting information that highlighted the integration of digital health technologies, such as EHR, telemedicine, mobile health applications, and data analytics. Fifth, the extracted information was compiled into a structured database or spreadsheet. The database included columns for university name, course code, course title, credit hours, and detailed course objectives and content. Sixth, the presence and structure of digital health and health informatics courses across different institutions were compared. Patterns, gaps, and variations in the integration of these subjects within the medical curricula were identified. Seventh, the recorded information was cross-checked with the original documents to ensure accuracy. If discrepancies were found or clarifications were needed, university departments were contacted for confirmation. Eighth, the findings were summarized in a comprehensive report, highlighting the extent of the integration of digital health and health informatics in medical curricula. Recommendations for improving the inclusion and depth of these subjects in medical education across Saudi Arabia were provided.

## Results

University and college overview

This study analyzed a total of 18 medical colleges and universities across Saudi Arabia, including a mix of public and private institutions. The sample represents a diverse range of academic offerings and institutional characteristics. Among the institutions, several are well-established, with histories dating back to the 1950s, while others are relatively new, reflecting the rapid expansion of medical education in the country. The study includes both large public universities, such as King Abdul Aziz University and King Saud University, as well as private institutions like Batterjee Medical College and Sulaiman Al Rajhi University. This comprehensive sample provides a broad view of the current state of medical education in Saudi Arabia, as detailed in Table 1.

**Table 1:** Overview of universities and colleges

University/college	Location	Type	Established	Overview
Batterjee Medical College (BMC)	Jeddah	Private	2005	Offers nine programs in medical and health sciences, with training opportunities in Ministry of Health hospitals and the Saudi German Hospital Group.
Dar Al Uloom University	Riyadh	Private	2008	Focuses on modern education, international quality, and academic accreditation.
Imam Abdulrahman bin Faisal University	Dammam	Public	1975	Known for pioneering postgraduate medical education and offering professional development programs.
King Abdul Aziz University	Jeddah	Public	1967	One of the largest and most prestigious universities in Saudi Arabia, offering a wide range of programs.
King Khalid University	Abha	Public	1998	Major universities offering various undergraduate and postgraduate programs.
Prince Sattam bin Abdulaziz University	Al-Kharj	Public	2009	Offers a variety of programs including medicine and health sciences.
Princess Nourah bint Abdulrahman University	Riyadh	Public	2008	Leading women's university established to advance women's education and contribute to various scientific fields.
Sulaiman Al Rajhi University	Al-Bukairiyah	Private	2009	Non-profit university with an academic partnership with Maastricht University, focusing on high-quality medical education and research.
University of Hail	Hail	Public	2005	Aligning with Saudi Vision 2030, aiming to excel in scientific research and academic education.
Al-Rayan Medical College	N/A	Private	N/A	Focuses on medical education with a comprehensive curriculum.
The College of Medicine at Al-Baha University	Al-Baha	Public	2014	Provides comprehensive medical education with a focus on foundational and clinical training.
VISION Medical College	Jeddah	Private	N/A	Offers a detailed medical program, focusing on foundational sciences and clinical practice.
Taibah University	Al-Madinah	Public	2003	Provides a detailed medical program with an emphasis on traditional medical education and integrated clinical practice.
University of Bisha	Bisha	Public	2014	Focuses on traditional medical education curriculum, including foundational sciences, clinical practice, and various medical systems and specialties.
University of Qassim	Qassim	Public	2004	Not available in the provided documents or on the website.
University of Al-Jouf	Al-Jouf	Public	2005	Not available in the provided documents or on the website.
University of Tabuk	Tabuk	Public	2006	Not available in the provided documents or on the website.
King Saud University	Riyadh	Public	1957	One of the oldest and most prestigious universities in Saudi Arabia, offering a wide range of programs.

Integration of digital health and health informatics courses

The analysis revealed that only a small number of the examined medical colleges offer dedicated courses on digital health or health informatics. Specifically, out of the 18 institutions examined, only three (Dar Al Uloom University, Princess Nourah bint Abdulrahman University, and the University of Hail) provide a specific course on medical informatics, each offering a two-credit-hour course covering essential topics such as EHR, clinical decision support (CDS), and healthcare innovations. The remaining institutions either lack such courses entirely or do not have available study plans that mention them. This indicates a significant gap in the integration of digital health and health informatics within the medical curricula across Saudi Arabia (as summarized in Table 2).

**Table 2:** Presence of digital health and health informatics courses N/A: not applicable, EHR: electronic health record, CPOE: computerized physician order entry, CDS: clinical decision support

University/college	Digital health/informatics courses (yes/no)	Specific courses mentioned	Credit hours	Objectives/content summary
Batterjee Medical College (BMC)	No	N/A	N/A	N/A
Dar Al Uloom University	Yes	MEDI301 - medical informatics	2	Introduction to information science in healthcare, covering EHR, CPOE, CDS, and minimizing medical errors.
Imam Abdulrahman bin Faisal University	No	N/A	N/A	N/A
King Abdul Aziz University	No	N/A	N/A	N/A
King Khalid University	No	N/A	N/A	N/A
Prince Sattam bin Abdulaziz University	No	N/A	N/A	N/A
Princess Nourah bint Abdulrahman University	Yes	CMI301 - medical informatics	2	Application of information science in healthcare, focusing on EHR, CDS, and evidence-based medicine.
Sulaiman Al Rajhi University	No	N/A	N/A	N/A
University of Hail	Yes	MIF 321 - medical informatics	2	Integration of IT in healthcare, including EHR, CDS, and healthcare innovations.
Al-Rayan Medical College	No	N/A	N/A	N/A
The College of Medicine at Al-Baha University	No	N/A	N/A	N/A
VISION Medical College	No	N/A	N/A	N/A
Taibah University	No	N/A	N/A	N/A
University of Bisha	No	N/A	N/A	N/A
University of Qassim	No (not available study plan)	N/A	N/A	N/A
University of Al-Jouf	No (not available study plan)	N/A	N/A	N/A
University of Tabuk	No (not available study plan)	N/A	N/A	N/A
King Saud University	No (not available study plan)	N/A	N/A	N/A

**Figure 1:**
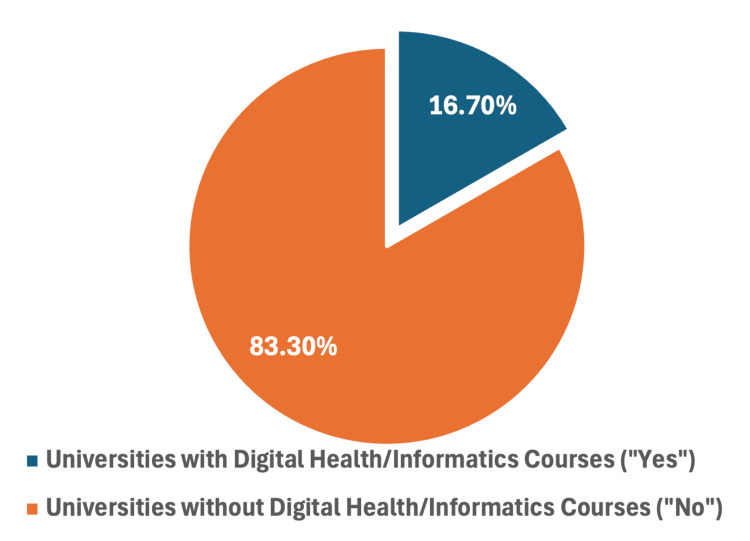
Distribution of universities with digital health/informatics courses

Figure 1 illustrates the distribution of universities that offer digital health and informatics courses. According to the analysis, only three out of 18 surveyed universities (16.7%) have integrated these courses into their curricula, while a substantial 15 out of 18 (83.3%) do not offer any courses specifically focused on digital health or health informatics (Figure 1).

The study identifies specific courses offered by only three out of the 18 investigated universities - Dar Al Uloom University, Princess Nourah bint Abdulrahman University, and University of Hail - that focus on digital health and health informatics. Dar Al Uloom University offers a course titled MEDI301 - Medical Informatics, a two-credit hour course that introduces students to information science in healthcare, covering EHR, computerized physician order entry (CPOE), CDS, and methods to minimize medical errors. Similarly, Princess Nourah bint Abdulrahman University provides a course titled CMI301 - Medical Informatics, also a two-credit hour course, focusing on the application of information science in healthcare and emphasizing EHR, CDS, and evidence-based medicine. University of Hail offers MIF 321 - Medical Informatics, a two-credit hour course that integrates information technology in healthcare, including EHR, CDS, and healthcare innovations (as summarized in Table 3).

**Table 3:** Detailed course information EHR: electronic health record, CPOE: computerized physician order entry, CDS: clinical decision support, IT: information technology

University/college	Course code	Course title	Credit hours	Objectives/content summary
Dar Al Uloom University	MEDI301	Medical informatics	2	Introduction to information science in healthcare, covering EHR, CPOE, CDS, and minimizing medical errors.
Princess Nourah University	CMI301	Medical informatics	2	Application of information science in healthcare, focusing on EHR, CDS, and evidence-based medicine.
University of Hail	MIF 321	Medical informatics	2	Integration of IT in healthcare, including EHR, CDS, and healthcare innovations.

## Discussion

Integration of digital health and health informatics in Saudi Arabian medical education

The findings of this study reveal a significant gap in the integration of digital health and health informatics courses across medical colleges in Saudi Arabia. Only 16.7% of the surveyed institutions offer dedicated courses in these areas, while the majority (83.3%) do not include them in their curricula. This indicates a need for substantial improvements to align with global trends and national healthcare objectives outlined in Saudi Arabia's Vision 2030.

The findings of this study reveal a significant gap in the integration of digital health and health informatics courses across medical colleges in Saudi Arabia. Only 16.7% of the surveyed institutions offer dedicated courses in these areas, while the majority (83.3%) do not include them in their curricula. This indicates a need for substantial improvements to align with global trends and national healthcare objectives outlined in Saudi Arabia's Vision 2030.

Global perspective

The integration of digital health and health informatics into medical education has seen varying levels of adoption globally, with several countries at the forefront of curriculum innovation. In the United States, institutions such as the University of California, San Francisco, have developed comprehensive programs that incorporate EHR training, clinical informatics, and telemedicine into the core medical curriculum [12]. A study by Shortliffe and Edward underscores that early exposure to health informatics tools significantly enhances medical students' abilities to manage patient data and utilize CDS systems effectively [13].

Similarly, European countries have taken a structured approach to embedding digital health into medical curricula. Institutions across these regions have implemented comprehensive programs and allocated dedicated resources to ensure that students are proficient in digital health technologies and their applications. Many US and European medical schools have successfully integrated EHR competencies and core biomedical informatics, including health information technology, into their curricula [14-17]. This structured approach contrasts sharply with the current state in Saudi Arabia, highlighting the need for the adoption of similar comprehensive strategies to enhance the country's medical education system.

Currently, digital health courses in many regions tend to be elective, often focusing on a single aspect of digital health and lacking robust evaluation mechanisms. This aligns with findings from Saudi Arabia, where there is a clear need for more structured and mandatory courses in this field. Innovative approaches, such as the use of online modules and Web 2.0 technologies in teaching medical informatics, have been described and could serve as valuable models for Saudi medical schools seeking to integrate digital health education more effectively [18-21].

Regional developments

In the Middle East, there has been a concerted effort to modernize medical education by incorporating digital health competencies. Medical students who received training in health informatics showed improved proficiency in managing patient data and using telemedicine platforms [4]. Similarly, in the United Arab Emirates, initiatives have been launched to integrate health informatics into medical curricula, recognizing its importance for the future of healthcare [22].

Compared to these regional advancements, Saudi Arabia's efforts appear minimal. While some progress has been made, the pace and extent of integration in Saudi medical colleges are insufficient to meet the rising demands of modern healthcare. The success of international summer schools on health informatics could serve as a model for Saudi Arabia to improve its integration efforts [23].

Furthermore, the importance of digital competence for future doctors is critical for the Saudi context [24]. Insights from health informatics education in Peru also provide valuable lessons on how emerging economies can tackle similar challenges [25]. These regional and global benchmarks underscore the urgent need for Saudi Arabia to enhance its digital health education.

National context: Saudi Arabia

Saudi Arabia's Vision 2030 emphasizes the modernization of its healthcare system, including the adoption of digital health technologies. The National eHealth Strategy aims to establish a comprehensive electronic health ecosystem, which necessitates the inclusion of digital health and health informatics education in medical training programs [24]. However, this study reveals that the integration of these subjects into the curricula of Saudi medical colleges is limited.

A survey of Saudi medical schools found that while some institutions have introduced courses on health informatics, the extent and depth of coverage vary widely. This finding is consistent with the results of the current study, which shows that only a few universities, such as Dar Al Uloom University, Princess Nourah bint Abdulrahman University, and University of Hail, offer specific courses on medical informatics. These courses cover essential topics such as EHR, CDS, and healthcare innovations, highlighting the importance of these areas in modern medical education [7].

The challenges faced by Saudi medical schools are echoed in studies from other contexts. Restrictions on nursing students' access to electronic health information hinder the development of informatics competency [25]. Similarly, the necessity of formal training in medical informatics to prepare students for the digital age has been emphasized [26].

Challenges and opportunities

The limited integration of digital health and health informatics courses presents several challenges, including a lack of qualified faculty, limited resources, and resistance to curriculum changes. Many students experience psychological pressure and a lack of understanding of the field, suggesting a need for better curriculum alignment and support structures [27].

However, there are also significant opportunities for improvement. Collaboration with international institutions, investment in faculty development, and leveraging advanced technologies can facilitate the integration of these critical areas into medical education. For example, partnerships with technology companies and healthcare organizations can provide valuable resources and expertise. Developing standardized digital health competencies and updating curricula to include ethical considerations of digital technologies can enhance the quality of medical education and better prepare students for the evolving healthcare landscape [9].

A five-tiered approach to integrating clinical informatics into undergraduate medical education could serve as a model for Saudi medical schools [28]. Additionally, the integration of health informatics into nursing education using a spiral learning approach ensures continuous reinforcement of key concepts [29,30]. These innovative methods highlight the potential for Saudi Arabia to adopt and adapt best practices from around the world.

Recommendations

Based on the findings of this study, several recommendations are proposed to improve the integration of digital health and health informatics into Saudi Arabian medical education. First, it is crucial to expand course offerings by introducing more courses dedicated to digital health and health informatics across all medical colleges. This will ensure that students gain comprehensive exposure to these critical fields. Additionally, developing standardized curricula that cover essential topics such as EHR, telemedicine, health data analytics, and mobile health applications can provide a uniform foundation of knowledge for all students. Investing in faculty development is also vital, as training and development programs can enhance the expertise of faculty members in digital health and health informatics, equipping them to deliver high-quality education. Leveraging technology and international collaborations can further enhance the integration of digital health and health informatics into medical education. Utilizing advanced technologies can provide innovative teaching tools, while partnerships with international institutions can bring valuable insights and resources. Finally, implementing support structures to alleviate psychological pressure on students and improve their understanding of the field is essential. These support structures can include counseling services, peer support groups, and academic resources tailored to the needs of students in digital health and health informatics programs.

Study limitations

This study provides important points about the integration of digital health and health informatics in the medical curricula of Saudi Arabian universities; however, several limitations should be acknowledged. The study includes a purposive sample of medical colleges, which may not fully represent the diversity of medical education programs across all universities in Saudi Arabia. Additionally, some institutions were not included due to the unavailability of detailed curricula or study plans, which could impact the comprehensiveness of the data. The study relied on publicly available information from university websites, academic catalogs, and direct communications with university departments. In some cases, the most recent or comprehensive documents may not have been accessible, potentially affecting the accuracy and completeness of the data. There is considerable variability in how universities document and present their curricula, and differences in terminology, structure, and detail levels may have impacted the consistency of data extraction and comparison across institutions. The study primarily focused on the presence and structure of courses related to digital health and health informatics without extensively evaluating other aspects of curriculum integration, such as practical training, extracurricular activities, or faculty expertise in these fields. Furthermore, the field of digital health and health informatics is rapidly evolving, and the study's findings represent a snapshot in time that may not fully capture recent developments or planned future enhancements in curricula. Finally, while the study analyzed course offerings and curriculum content, it did not include qualitative data from students and faculty about their experiences, perceptions, and challenges related to digital health and health informatics education. Such insights could provide a more comprehensive understanding of the integration and effectiveness of these courses. Differences in institutional priorities, resources, and strategic goals may also influence the integration of digital health and health informatics in medical curricula, and these contextual factors were not fully explored in this study.

## Conclusions

After conducting this study, it is evident that there is a need for a more standardized and comprehensive approach to integrating digital health and health/medical informatics into the medical curricula of Saudi Arabian universities. Future doctors must be fully aware of the significant potential brought by these innovative technologies that exist and will widely arise in healthcare settings. Addressing these gaps will better prepare medical graduates for the modern, technology-driven healthcare environment and align with the national objectives of Vision 2030. By incorporating these recommendations, Saudi medical colleges can enhance their educational frameworks, ensuring that future healthcare professionals are well-equipped to navigate and innovate in the important field of digital health.
